# Ultrasound-guided Bedside Core Needle Biopsy: A Hospitalist Procedure Team’s Experience

**DOI:** 10.7759/cureus.3817

**Published:** 2019-01-03

**Authors:** Sanjay A Patel, Krzysztof Pierko, Ricardo Franco-Sadud

**Affiliations:** 1 Internal Medicine, John H. Stroger, Jr. Hospital of Cook County, Chicago, USA; 2 Internal Medicine, NCH Healthcare System, Naples, USA

**Keywords:** core biopsy, needle biopsy, cost savings, bedside procedures, point of care ultrasound

## Abstract

Tissue pathology is integral for the diagnosis of various conditions, especially malignancy. Traditionally, biopsy procedures, including core needle biopsy (CNB), are performed by surgeons or radiologists. With the increasing utilization of point of care ultrasound (POCUS) skills and competence in bedside procedures by general internists, CNB can be safely moved to the patient’s bedside with maintained accuracy and increased cost savings compared to traditional procedural methods. We aim to review the experience of our hospitalist-run medical procedure service in performing these ultrasound-guided procedures at the bedside.

## Introduction

Biopsy of a nodule, mass or lymph node is the gold standard for diagnosis of a variety of conditions, especially malignancy. Tissue sampling can be done via one of three methods—fine needle aspiration (FNA), core needle biopsy (CNB) or open surgical biopsy. Choosing a method is dependent upon the suspected pathology, procedural safety, patient factors and preference. FNA and CNB allow for less invasive procedures, lesser healthcare costs and shorter recovery times [1-3]. Pathology from open biopsy has historically provided better diagnostic yield, but FNA and CNB have gained traction as reasonable alternatives without sacrificing diagnostic outcomes [1,4-7]. Across a range of pathologies, the yield of CNB (81-95% sensitivity, 80-100% specificity, 93-95% accuracy) compared to FNA (63-80% sensitivity, 70-80% specificity, 80% accuracy) has made it a more preferable initial test of choice [8-10]. CNB is less prone to sampling errors and allows for better characterization of the target lesion compared to FNA, often requiring less follow-up procedures [5,11,12]. The addition of procedural ultrasound guidance provides safety benefit along with characterization of target lesions, contributing more pre-procedural bedside clinical information than other biopsy modalities [13,14].

Biopsy procedures have historically been a duty of surgeons, radiologists and subspecialists. They are usually performed in special procedure rooms, interventional radiology suites or operating rooms. Along with physical location comes the added costs and resources of nursing staff, procedural sedation and monitoring, transportation and equipment. Meanwhile, general internists, particularly hospitalists, are increasingly becoming familiar and competent with bedside procedures [15,16]. Combined with the advent of hospitalist-run bedside procedure services across the nation, a growing interest has sprouted in ultrasound-guided bedside procedures [17,18]. Ultrasound machines have become more portable over time and the skillset of the general internist has increased accordingly. This combination aims to increase healthcare efficiency while decreasing overall cost. A growing body of literature supports the safety of needle biopsy being done at the bedside with sonographic guidance [19]. Training for these procedures can be incorporated as part of the instruction and development curriculum for a variety of other bedside procedures and point-of-care ultrasound applications.

CNB is not typically within the scope of existing bedside procedure teams. We aim here to review our team’s pilot experience in a large urban safety-net tertiary center. Particular interest is focused on technical safety, cost savings and future lessons for improvement in performing bedside ultrasound-guided biopsies by general internists.

## Materials and methods

Our medical procedure service (MPS) is comprised of five attending hospitalists (proceduralists) and one nurse clinician trained in point-of-care ultrasound and basic bedside medical procedures (paracentesis, thoracentesis, lumbar puncture and vascular access). Since its inception in 2006, an average of 2200 procedures are completed yearly. All procedures are requested via computerized physician order entry to allow accurate record keeping of performance and quality measures.

From January, 2015 to June, 2017, the MPS received 73 requests for bedside CNB. Based upon patient factors, equipment availability, lesion size and provider logistics, 60 biopsies have been performed. Over this time, three faculty self-volunteered to train and perform these procedures along with lymph node ultrasound, with two of those three faculty performing the majority (55/60, 92%) of the procedures. Thirty-five biopsies were performed with a 14-gauge, automatic spring-loaded biopsy device. The remaining 25 biopsies were performed with an 18-gauge device. The decision of core needle size was left solely to the provider based upon lesion size or availability of equipment. All procedures were done with sterile equipment and technique, and all were performed with dynamic real-time ultrasound guidance using local anesthesia alone.

**Figure 1:**
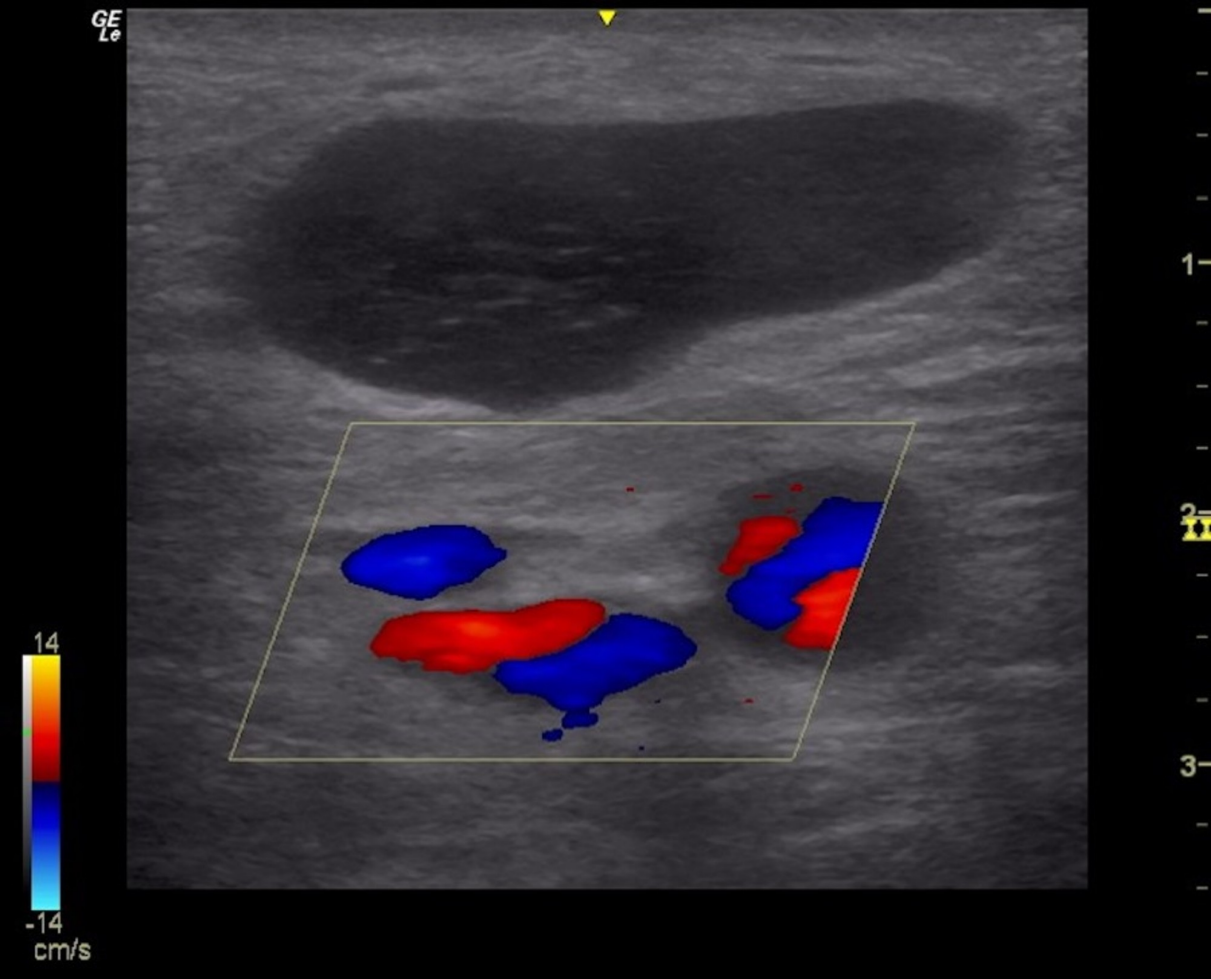
Metastatic lymph node with surrounding vasculature.

The sole eligibility criteria for the consideration of a biopsy was that a lesion be superficial and either readily palpable or visible to the physician. The decision to move forward with a CNB request was dependent upon the proceduralist’s comfort with dynamic ultrasound imaging of the lesion. Reasons not to perform the procedure included inadequate lesion size, safety concerns (Figure 1), lack of appropriate equipment or patient refusal. The excluded requests were referred to the appropriate subspecialist for consideration.

## Results

The total number of samples obtained was 197 over 60 biopsies, an average of 3.3 samples per procedure (Table 1). The average size of lesions was 3.3 cm, with a range of 1.5 to 10 cm. Eighty-three percent (50/60) of the biopsies were performed on the same day, with an average request-to-procedure time of 3.5 hours. The remaining 10 were done the next working day, with the reason for delay in all cases being that the procedure was requested late in the day or over a weekend. The pathology submitted from all biopsies yielded an initial definitive diagnosis in all but four cases. All four cases (4/60, 6.7%) had an initial histologic diagnosis of reactive adenopathy. Follow-up excisional biopsy in all cases, driven by clinical suspicion for malignancy, supported the initial CNB pathology. This correlated to an actionable diagnostic yield of 93.3%, similar to reported literature [7-9,11]. Considering the final excisional biopsy results of the aforementioned four procedures, the retrospective diagnostic accuracy of bedside biopsies was 100%.

All but 21.7% (13/60) of cases returned a diagnosis of malignancy. Four demonstrated reactive adenopathy as discussed already. Of the remaining eight cases, one revealed non-caseating granulomas and lymphadenitis (final diagnosis of sarcoidosis was made based upon clinical presentation and pathology). Another four revealed caseating granulomas where the final diagnosis of tuberculous lymphadenitis was made. Two cases revealed a benign tumor—a fibrous histiocytoma and a lipoma. One case revealed a dense methicillin-resistant *staphylococcus aureus* (MRSA) panniculitis that was treated successfully with antibiotic therapy. The final case revealed actinomycosis, treated successfully with antibiotics upon identification from tissue culture.

Malignancy was the most common diagnosis, with 78.3% (47/60) of procedures yielded an actionable diagnosis of cancer. Of this total, 24/47 (51%) were of the hematologic variety and 23/47 (49%) were of the solid tumor variety. None of these cases required subsequent nodal tissue sampling as enough information was obtained from the CNB for definitive diagnosis. The most common of these diagnoses was non-Hodgkin lymphoma. Of the solid malignancies, the pathologies ranged across a spectrum of tumor types (Table 1).

**Table 1:** Diagnosis by pathology (n = 60).

Non-Malignant (n = 13)	Hematologic Malignancy (n = 24)	Solid Tumor Malignancy (n = 23)
Actinomycosis (1)	Chronic Lymphocytic Leukemia (1)	Colorectal Adenocarcinoma (3)
Fibrous Histiocytoma (1)	Hodgkin Lymphoma (2)	Gynecologic Adenocarcinoma (4)
Lipoma (1)	Non-Hodgkin Lymphoma (15)	Head & Neck Squamous Cell (2)
Sarcoidosis (1)	Plasmacytoma (2)	Lung Adenocarcinoma (4)
Tuberculosis (4)	T-Cell Lymphoma (3)	Lung Squamous Cell (4)
Panniculitis (1)		Lung Small Cell (1)
Reactive Lymphadenopathy (4)		Papillary Thyroid (1)
		Prostate Adenocarcinoma (2)
		Spindle Cell Carcinoma (1)
		Poorly Differentiated Adenocarcinoma (1)

One minor complication occurred in the group. A patient, who was on dual-antiplatelet therapy, had mild bleeding (20 ml) from the puncture site that resolved within minutes following application of direct pressure. This accounted for 1.7% (1/60) of the patient cohort, similar to described rates of hemorrhage in the literature—the most well-described procedural risk [7,19,20]. Post-procedural ultrasonography did not reveal any evidence of hematoma. The overall group had average normal coagulation and hematologic parameters.

## Discussion

CNB of superficial lesions, in particular lymph nodes, at the bedside is a promising alternative to traditional CNB performed by subspecialists or traditional open biopsy procedures. In our patient review, diagnostic accuracy is comparable, if not better, to that already described in the literature [8,10,16]. Though our model exclusively examined inpatients, we believe there is generalizability to the outpatient setting—whereby costs, time-to-diagnosis and specialty referrals could further decrease. Similar to renal and liver biopsy, bedside CNB of superficial lesions with sonographic localization is a skill that is learnable [19,21,22]. Training time is minimal compared to open biopsy methods, with the advantage of increased convenience, decreased cost and improved healthcare efficiency—all while maintaining similar rates of diagnostic accuracy and complication rates. Bedside CNB with ultrasound guidance should be incorporated into the general internist’s armament of diagnostic testing.

While our study demonstrates many advantages, we recognize certain limitations. Prerequisite for biopsy consideration was that the lesion be readily visible or palpable. Assuredly, this introduced a selection bias, with the likelihood of obtaining successful tissue with minimal complications being higher with larger and more superficial lesions [23,24]. However, this frame of thinking could apply to any specialist-performed procedure, including interventional radiology or surgery. An essential element of moving forward with any procedure, including CNB, is the technical feasibility of that procedure. It naturally follows that the more feasible a procedure is, the more successful—in this case measured by accuracy and complications. It would likewise be naive to ignore how the experience of the provider would play into the technical aspects of the procedure. We propose that utilizing CNB at the bedside be reserved for more readily accessible lesions, at least initially. Better-defined roles and responsibilities between specialists and general internists are needed.

Training required, though minimal, may also be a limiting factor in broader implementation of bedside CNB by general internists. One potential limiting factor is accessibility to ultrasound, which is institution-specific. In our example above, the procedures were carried out by trained proceduralists who are staff in inpatient MPS. They readily have access to an ultrasound machine for imaging guidance that was of no added overall capital cost. If not readily available, institutional purchase of an inexpensive point-of-care ultrasound machine may be of interest, especially with increasing popularity of imaging-guided procedures and point-of-care ultrasound applications. Universally defining procedural competency has been historically deficient, and assessing expertise may be difficult [25]. Evaluation processes have been proposed for other high-volume procedures performed by internists—including central line insertion, lumbar puncture, thoracentesis and paracentesis. Guidelines are also anticipated soon from the Society of Hospital Medicine on training and credentialing in such procedures and point of care ultrasonography by hospitalists [26,27]. Yet, for bedside ultrasound-guided CNB, no such defined training parameters exist. Additional operator bias was likely present, being that the proceduralists were self-selected and likely to be skilled operators compared to any non-procedure oriented physician.

Although we did not discreetly measure cost savings, we presume there to be a significant decrease in patient and hospital costs for such provided services. A reduced need for patient transport, consultations for the sake of a procedure may impact the length of hospital stay—especially provided biopsies can be performed on the same day as in our model. As mentioned, nearly 75% of biopsies were done on the same day they were requested which assisted in hospital throughput. Assuming costs hold true from existing data of other procedures moved to the bedside, i.e. peripherally inserted central catheter (PICC), a minimum cost of the radiology suite (range $300-2000) can be saved [28,29]. Multiplied across our procedures would have saved between $18,000 and $120,000. This is a conservative estimate, excluding potential savings from the length of stay, transportation, nursing staff and equipment and medication costs. Across a range of institutions, this figure could presumably balloon into the hundreds of thousands of dollars when applied to the United States healthcare system. We theorize that by performing biopsies as an inpatient, we also decreased the time to diagnosis and prevented delays in outpatient referrals and treatment. Patient satisfaction with a bedside CNB, similar to other procedures, may also be preferable, but directly unmeasured [30].

## Conclusions

Bedside CNB by internists is a safe and effective alternative to traditional open biopsy procedures and specialist performed CNB. As evidenced by the series described above, it is learnable by a variety of physicians across healthcare specialties. The likeliest benefit is for general medical services—including, but not limited to, pediatrics, internal medicine, hospital medicine and family medicine providers. With the growing use and interest in bedside ultrasound, an equally growing interest and use of CNB can be justified to improve healthcare efficiency and cost. Ultrasound-guided CNB can be added to armament of skills available to internists. A definitive cost assessment is needed along with a standardized training model. In conclusion, we believe moving these procedures to the bedside by internists would improve the overall delivery of care to patients.
